# Prenatal cytogenomic identification and molecular refinement of compound heterozygous *STRC* deletion breakpoints

**DOI:** 10.1002/mgg3.806

**Published:** 2019-06-19

**Authors:** Lisong Shi, Yan Bai, Yara Kharbutli, Andrea M. Oza, Sami S. Amr, Lisa Edelmann, Lakshmi Mehta, Stuart A. Scott

**Affiliations:** ^1^ Department of Genetics and Genomic Sciences Icahn School of Medicine at Mount Sinai New York City New York; ^2^ Sema4, a Mount Sinai Venture Stamford Connecticut; ^3^ Laboratory for Molecular Medicine Partners Personalized Medicine Cambridge Massachusetts

**Keywords:** allele‐specific PCR, *CATSPER2*, chromosomal microarray, copy number variation, deafness‐infertility syndrome, prenatal diagnosis, *STRC*

## Abstract

Here, we report the prenatal detection of a compound heterozygous deletion at chromosome 15q15.3 by clinical chromosomal microarray (CMA) testing that included the *CATSPER2* male infertility gene. However, given the low resolution of CMA at this homologous locus, it was unclear if the neighboring *STRC* hearing loss gene was also affected. Therefore, we developed a novel allele‐specific PCR strategy, which narrowed the proximal breakpoint of the maternally inherited deletion to a 310 bp interval that was 440 bp upstream from the *STRC* transcription start site.

To The Editor:

Hearing loss is the most common neurosensory disorder in humans, affecting ~466 million individuals worldwide (https://www.who.int/news-room/fact-sheets/detail/deafness-and-hearing-loss). Approximately 70% of affected individuals have nonsyndromic hearing loss (NSHL), which currently has over 115 recessive, dominant and/or X‐linked genes implicated in its molecular pathogenesis (https://hereditaryhearingloss.org/). The DFNB1 locus (*GJB2* and *GJB6*) accounts for ~50% of severe to profound congenital NSHL cases, followed by *USH2A*, *STRC*, and *MYO15A* (Francey et al., 2012; Vona et al., 2015; Zazo Seco et al., 2016). Pathogenic recessive sequence variants in *STRC* were first reported in 2001 and this gene is now estimated to account for ~5%–6% of all congenital sensorineural hearing loss cases (Francey et al., 2012; Markova et al., 2018; Shearer et al., 2014; Sloan‐Heggen et al., 2016; Vona et al., 2015); however, this could be an underestimate given the variable *STRC* allele frequencies between ethnicities (Sloan‐Heggen et al., 2016) and the ongoing appreciation of clinically relevant copy number variation at the *STRC* locus.

The *STRC* gene encodes stereocilin, which plays an important role in the outer hair cell stereocilia of the inner ear. However, a highly homologous (>99%) distal pseudogene (*pSTRC*) at chromosome 15q15.3 makes it very challenging to specifically interrogate *STRC* by most molecular assays. The directly oriented ~100 kb segmental duplications that encompass *STRC* and *pSTRC* can also act as substrates for nonallelic homologous recombination, which leads to the formation of recessive *STRC* deletions as well as the reciprocal duplications (Mandelker et al., 2014; Shearer et al., 2014; Sloan‐Heggen et al., 2016; Vona et al., 2015). In addition, *STRC* deletions that include the neighboring *CATSPER2* gene are a recessive cause of deafness‐infertility syndrome (prevalence of ~1 in 40,000; Avidan et al., 2003; Hoppman et al., 2013; Karger et al., 2017; Knijnenburg et al., 2009; Zhang et al., 2007), which is characterized by early onset deafness in males and females, but infertility exclusively in males, as CATSPER2 is required for sperm motility.

Importantly, the extensive homology across the *STRC* and *CATSPER2* region results in only a small number of nucleotides that differentiate Mendelian disease genes from their corresponding pseudogenes. As such, molecular hybridization‐based copy number detection platforms (e.g., multiplex ligation‐dependent probe amplification [MLPA], droplet digital PCR [ddPCR], chromosomal microarray [CMA]) have very low resolution at this locus, particularly across the 28 exons of *STRC* (Francey et al., 2012; Markova et al., 2018). Here, we report the prenatal detection of a compound heterozygous deletion at chromosome 15q15.3 that included *CATSPER2*; however, it was unclear if *STRC* was affected by CMA testing, which subsequently was elucidated by a novel allele‐specific PCR (AS‐PCR) strategy.

A 39‐year‐old female was referred for prenatal genetic testing for advanced maternal age and a previous pregnancy with trisomy 22. G‐banded chromosome analysis of the chorionic villus sample indicated a normal female karyotype; however, prenatal CMA testing (4x180K CGH + SNP; Agilent Technologies, Santa Clara, CA) detected an apparently homozygous 33.7 kb deletion of chromosome 15q15.3 that included *CATSPER2* [reported as arr[GRCh37] 15q15.3(43916972_43950720)x0] (Figure 1). The homozygous loss of *CATSPER2* was confirmed by a higher density microarray (244K, Agilent Technologies) and ddPCR (Amr et al., 2018) at intron 7 and exon 7 of *CATSPER2*; however, parental CMA testing and ddPCR at introns 25 and 23 of *STRC* indicated that the paternal chromosome 15q15.3 deletion included *STRC* whereas the maternal chromosome 15q15.3 deletion was inconclusive at the 5’ region of *STRC* due to the absence of copy number probes from exons 1 to intron 22 of *STRC* and ~6.0 kb upstream of the transcription start site. Due to these inconclusive maternal results, accurate counseling about potential hearing loss in the child could not be provided at that time.

**Figure 1:**
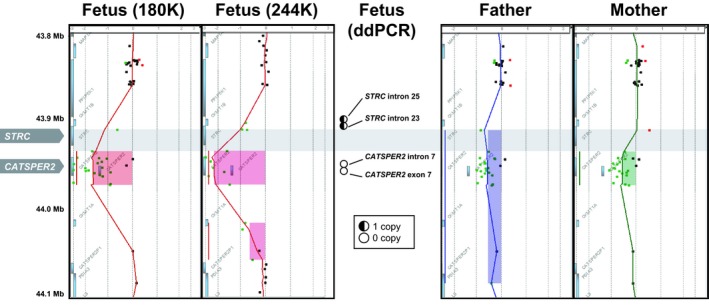
Chromosomal microarray (CMA) results illustrating an apparently homozygous 33.74 kb deletion on chromosome 15q15.3 in the fetus, which was confirmed by 244K CMA and ddPCR. Parental follow‐up by CMA indicated that both parents were heterozygous carriers of deletions at this region. Gray horizontal bar highlights the region overlapping the *STRC* gene with minimal probe coverage

To further resolve the proximal breakpoint of the maternal deletion, a novel two‐tiered AS‐PCR strategy was developed. The first round of AS‐PCR had 14 unique amplicons across a ~110 kb interval covering both *STRC* (NM_153700) and *CATSPER2* (NM_172095). Primers were designed using Primer 3 web version 4.0 (sequences available upon request; http://bioinfo.ut.ee/primer3/) and all amplicons were examined on 1% agarose gels. As expected, all 14 specific targets were successfully amplified from parental DNA given the presence of at least one intact copy in both parents; however, AS‐PCR amplicons 8–14 were absent in fetal DNA (Figure 2) confirming the homozygous *CATSPER2* deletion detected by prenatal CMA testing. These results narrowed the maternal deletion to a ~1.6 kb region between AS‐PCR amplicons 7 and 8, which included exons 1 and 2 of *STRC* (Figure 2). A single nucleotide subsequently was identified in exon 2 of *STRC* that distinguished this 1.6 kb region from *pSTRC*, and this position was used as a 5' oligonucleotide anchor (positive strand) for a second round of AS‐PCR of seven separate amplicons (163–1623 bp) using nonunique 3' primers (sequences available upon request; Figure 2). Successful amplification of AS‐PCR amplicons 1–6 and the absence of amplicon 7 in the fetus narrowed the proximal breakpoint of the maternal deletion to a 310 bp interval that was 440 bp upstream from the *STRC* transcription start site. These results indicated that the fetus carried one intact copy of *STRC* (Figure 2). In addition, CMA testing on DNA from an unaffected child of the parents detected a heterozygous 55.1 kb deletion that included exons 1–22 of *STRC* and the *CATSPER2* gene [reported as arr[GRCh37] 15q15.3(43895633_43950720)x1], which was considered paternally inherited.

**Figure 2:**
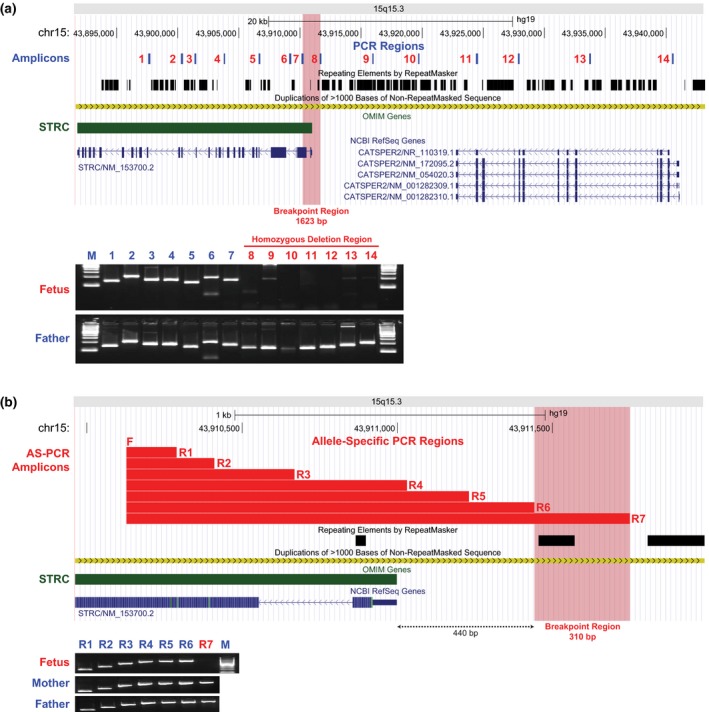
(a) The first round of allele‐specific PCR (AS‐PCR) indicated that the 5' maternal deletion breakpoint was located in a ~1.6 kb region between amplicon 7 and 8. (b) The second AS‐PCR revealed that the maternal deletion 5' breakpoint was located within a ~300 bp region at ~440 bp upstream of the *STRC* gene

Given these test results, the couple was provided reassurance regarding hearing loss. The baby girl was born naturally at 39.5 weeks after an uncomplicated pregnancy and passed the newborn hearing screen. Importantly, no concerns have been noted on clinical follow‐up, and at 4 years of age, she currently is thriving with excellent hearing and normal development.

Although many different molecular platforms are used to detect *STRC* and *CATSPER2* deletions (e.g., MLPA, ddPCR, qPCR, CMA), all currently available clinical assays have low resolution across this region, which is considered one of several disease regions in the human genome that is not amenable to direct interrogation by hybridization‐based assays and/or short‐read sequencing (Baux et al., 2017; Mandelker et al., 2016). There are ~56 divergent nucleotides between exons 16–28 of *STRC* and *pSTRC*; however, exons 1–15 have >99% sequence homology. The paucity of CMA probes across the *STRC* gene is particularly challenging when interpreting prenatal CMA results as breakpoints, affected gene content, and predicted phenotype are uncertain. As such, our novel AS‐PCR strategy helped to elucidate the breakpoint of an otherwise uncertain deletion that potentially included *STRC* in addition to *CATSPER2*. Taken together, these data highlight important challenges in the prenatal detection of homologous disease gene regions and underscore the need for higher resolution molecular methods (e.g., long‐read sequencing) across selected disease genes to better inform phenotype prediction and prenatal genetic counseling.

## CONFLICT OF INTEREST

L.S., L.E., and S.A.S. are paid employees of Sema4, a Mount Sinai venture, Stamford, CT.

## Data Availability

The datasets generated during and/or analyzed during the current study are not publicly available due to these data being derived from clinical genetic testing; however, deidentified data may be available from the corresponding author on reasonable request.
